# Book Reviews

**Published:** 1900-02

**Authors:** 


THE TREATMENT OF PELVIC INFLAMMATION THROUGH THE VAGINA. By William R. Pryor, M.D., New York. With 110 illustrations. Price $2.00, net. W. B. Saunders, Philadelphia, 1899.

This work by one of its foremost advocates is a plea for the vaginal route in the treatment of various inflammatory conditions of the female pelvic organs and the results of such inflammations. The subjects covered are endometritis, salpingitis, pelvic peritonitis, inflammatory diseases of the ovaries, diseases of the broad ligament, diffuse pelvic suppuration. Then follow well illustrated and specific descriptions of the various operative procedures, with account of after-treatment, possible accidents and the like. The book forcibly sets forth the trend of modern teaching and will doubtless be well received.

---

BACTERIOLOGY IN MEDICINE AND SURGERY. A Practical Manual for Physicians, Health Officers and Students. By Wm. Hallock Park, M.D., Associate Professor of Bacteriology and Hygiene, University and Bellevue Hospital Medical College, and Assistant Director of the Research Bacteriological Laboratories of The Department of Health, City of New York. Assisted by A. R. Guerard, M.D., Assistant Bacteriologist, Department of Health, City of New York. Illustrated with eighty-seven engravings and two color plates. Lea Brothers & Co., New York and Philadelphia.

Park is well known in connection with the bacteriological work of the New York Board of Health. His book is the outcome of considerable original investigation. Bacteriology is constantly becoming more important in relation to medicine and surgery, and an acquaintance more or less close with this difficult science is essential to the equipment of the practitioner of to-day.

The book covers the ground admirably and will more than likely be adopted as text-book in many of the colleges.

---

TEXT-BOOK OF THE PRACTICE OF MEDICINE. By James M. Anders, M.D., Professor of Practice in Medico-Chirurgical College of Philadelphia. Third edition. Completely illustrated. Published by W. B. Saunders, Philadelphia, 1899.

Anders's Practice is already very well known and well liked. It is embraced in one volume and can be recommended as a complete and useful guide. The treatment of disease is very fully considered —a feature much appreciated by one in active practice.

HYGIENE OF TRANSMISSIBLE DISEASES. By A. C. Abbott, M.D. Price $2.00. W. B. Saunders, Philadelphia, 1899.

This work treats briefly of the prophylaxis and general hygienic consideration of the communicable diseases and is to be classed as a contribution to preventive medicine.

The book deals with causation, the study of causation, modes of dissemination and prevention of transmissible diseases. The work is well and clearly written.

---

COMPEND OF THE PRACTICE OF MEDICINE. By Daniel E. Hughes, M.D. Sixth edition. Published by P. Blakiston's Son & Co., Philadelphia. Price $2.25.

This is a most convenient and serviceable compend on the practice of medicine. The subject-matter is given in condensed form, but is sufficiently full to offer help to the busy doctor. The sixth edition has been revised and brought thoroughly up to date.

